# Towards a Sustainable Future: Advancing an Integrated Approach for the Recycling and Valorization of Agricultural Plastics

**DOI:** 10.3390/polym15234529

**Published:** 2023-11-25

**Authors:** Susana Filipe, Paulo Mira Mourão, Nazaré Couto, Davide Tranchida

**Affiliations:** 1 MED—Mediterranean Institute for Agriculture, Environment and Development & CHANGE—Global Change and Sustainability Institute, Institute for Advanced Studies and Research, Universidade de Évora, Pólo da Mitra, Ap. 94, 7006-554 Évora, Portugal; 2 MED—Mediterranean Institute for Agriculture, Environment and Development & CHANGE—Global Change and Sustainability Institute, Institute for Advanced Studies and Research, Departamento de Química e Bioquímica, Universidade de Évora, Pólo da Mitra, Ap. 94, 7006-554 Évora, Portugal; pamm@uevora.pt; 3 CENSE—Center for Environmental and Sustainability Research & CHANGE—Global Change and Sustainability Institute, Department of Environmental Sciences and Engineering, NOVA School of Science and Technology, NOVA University Lisbon, 2829-516 Caparica, Portugal; md.couto@fct.unl.pt; 4 Competence Center Advanced Polymer Characterisation, Borealis GmbH, Sankt Peter Strasse 25, 4021 Linz, Austria; davide.tranchida@borealisgroup.com

**Keywords:** plastics, agriculture, governance, public policies, wastes, recycling, environment, valorization

## Abstract

Plastic pollution has become a pressing environmental issue. The agricultural sector, in particular, is a significant contributor to this problem, given the widespread use of plastics in farming practices and a lack of and/or use of inefficient approaches for the recycling and valorization of agricultural plastic waste. This has resulted in the accumulation of these residues in landfills and/or their improper disposal, which has exacerbated their environmental impact, leading to negative consequences on soil, water, and ecosystems. This work provides an overview on the current methodologies available to address the challenges associated with inadequate management of agricultural plastics and highlights the need for a comprehensive and systematic methodology, involving material development, polymer processing, waste collection, sorting, and valorization. It emphasizes the importance of collaboration between polymer producers, polymer manufacturers, farmers, policymakers, waste management companies, and recyclers to develop effective, technical, and economically viable recycling and valorization schemes. This paper addresses gaps and provides guidance on possible solutions, specifically polymer development, policy instruments, regulatory frameworks, collection schemes, and the technical approaches required for the adequate valorization of agricultural plastic waste. Furthermore, it highlights the associated barriers and benefits of the different presented approaches. It also aims to promote awareness on agricultural plastic waste and provide guidance on the best approaches to reduce its environmental impact.

## 1. Introduction

### 1.1. Key Role of Plastics in Global Agriculture

The importance of plastic use in agriculture is undeniable, given its association with increasing food safety and quality, improving crop protection, water management, and ultimately, reducing the agriculture sector’s environmental footprint. The use of plastic within agriculture plays a vital role in pest control, pesticide emission, as well as a reduction in fertilizer and the use of phytopharmaceutical products, thus enabling the development of more sustainable and efficient agriculture practices.

Plastics in agriculture are applied within a variety of applications, which include but are not limited to greenhouses, mulching, low tunnels, silage, irrigation, transportation, and storage [1,2,3,4,5,6]. Greenhouse applications provide adequate control of temperature and moisture and protect crops from harmful environmental conditions and pests. Furthermore, the use of greenhouses enables controlled exposure of crops to sunlight and extreme cold, thus allowing an extension of the growing season and overall productivity increase. Mulching applications adequately enable better crop management through improved temperature control, moisture retention, nutrient management, enhanced weed control, and avoidance of contact between the plants and the ground. Silage has been used for the appropriate storage of grain and straw during winter, and it is essential to guarantee the production and availability of healthy, long lasting, and nutritious fodder and facilitate easier transportation of grain and straw.

Another important application of plastics in agriculture is the one associated with the use of irrigation systems, e.g., pipes, fittings, spray cones, and drip lines. These systems allow for an optimization of water use through either direct use or precision irrigation.

Other agriculture areas requiring the use of plastics include collection, storage, and transportation of vegetables, fruits, and other products. These approaches lead to the reduction of food loss during post-harvest transportation and storage. Plastic packaging of different forms and varieties are also used in agriculture. Examples include packaging products, bottles, and other containers for fertilizers and phytopharmaceutical products.

### 1.2. Issues Related to Inefficient Management of Plastic Waste (Soil and Water Properties)

As previously stated, plastic is widely employed in agriculture, with significant benefits including food security improvement, improved sustainability, and resource management. However, ineffective and/or inexistent collection and valorization schemes for agricultural plastic waste might lead to serious environmental problems in the long-term.

Degradation of agricultural plastic materials, in particular films, occurs as a consequence of exposure to solar radiation, rain, wind, extreme weather conditions such as temperature and humidity, and ultimately, exposure to chemicals, soil, stones, etc. In the absence of adequate plastic waste collection and management schemes, plastic waste might end up abandoned in fields, either buried or simply disposed of. Plastics themselves and contaminants (e.g., pesticides, phytopharmaceutical products adsorbed within the surface of plastics, etc), can end up as contaminants for soil, water, food, and ecosystems. A lack of appropriate waste management approaches for agricultural plastic waste can result in the abandonment and accumulation of plastic residues, which will degrade into smaller particles (microplastics) which, together with plastic additives, will accumulate in the soil. The accumulation of microplastics in the soil will affect soil health, specifically organisms such as earthworms, mycorrhizal fungi, and the overall soil microbiome, thus affecting soil health and productivity. In the long-term, this can lead to reduced agriculture productivity, thus threatening food security. In more extreme cases, migration of plastic fragments and microplastics through water runoff can further contaminate water streams, such as lakes, rivers, and oceans.

The migration of microplastics might also affect ecosystems, with negative effects on fauna and flora health. Chae et al. have reported the transfer from nano-plastic from soil to cup bean leaves, and from these to the snails via ingestion of the contaminated leaves [7]. The transfer and accumulation in food chains and exposure to humans via trophic transfer, inhalation, or even ingestion has been associated with infertility, blood vessel impediment, and other health problems [8].

The dimensions and impacts of improper agricultural plastic waste management significantly depend on the type of polymer used and the final product. The typical lifespan of agricultural plastics ranges from 5 to 60 months, depending on the type of plastic and its application. Greenhouse films, which constitute the application using the highest volume of plastics, have a typical lifetime ranging from 40 to 50 months. Irrigation tubes and mulch films, which represent the second- and third-highest volume plastic applications in agriculture, have lifespans of approximately 25 and 5 months, respectively [2].

Over the years, appropriate polymer design and additivation have led to an improved lifespan of agricultural plastics. Consequently, new polymers used in agriculture have converged to application solutions which require less material and/or positively affect the mechanical and environmental strength of the materials. Despite the R&D efforts mentioned above to improve plastic properties and increase their lifespan, the overall growth projections for agriculture and the consequent expected increase in plastic use in agriculture requires urgent action for plastic waste management. There is a need for the development of integrated models, policies, and mechanisms combining different inputs and involving different stakeholders from the entire plastic value chain. An appropriate holistic solution, ensuring a balanced use of plastic in agriculture and adequate cradle-to-grave agricultural plastic management, should consider the input from different areas, including material development, material processing and usage, end-of-life product dismantling, waste collection and recycling, waste valorization, soil monitoring, and soil restoration/remediation. An integrated approach for recycling and valorization of agricultural plastics will also benefit from the development of adequate policy tools and instruments, the involvement of the civil society and end users, and an overall improvement in knowledge and literacy.

## 2. General Overview of Plastics used in Agriculture

### 2.1. Plastics Used in Agriculture and Their Main Applications

The typical plastics used in agriculture consist of biobased and fossil-based polymers. Fossil-based polymers include non-biodegradable polyolefins such as linear low-density polyethylene (LLDPE), linear density polyethylene (LDPE), high-density polyethylene (HDPE), polypropylene (PP), expanded polystyrene (EPS), ethylene–vinyl acetate copolymers (EVA), and less frequently, poly-vinyl chloride (PVC), polyethylene terephthalate (PET), polycarbonate (PC), and poly-methyl-methacrylate (PMMA).

Polyolefins, such as PP, HDPE, and LDPE, constitute most of the plastics used in agriculture applications. Film applications for greenhouses, bale silage, mulching, and coatings typically use LDPE. Thicker films and other rigid applications, such as bales and other products including chemical containers and irrigation pipes, are often made out of HDPE. PP is typically used within protective woven and nonwoven films, fertilizer bags, tree guards, bulk containers, and polymer crates. Table 1 summarizes some examples of plastics and their applications within agriculture-related activities.

Biopolymers have been developed and have emerged as a possible alternative to fossil-based polymers. Examples of biobased and biodegradable polymers include starch, polylactic, polybutylene adipate terephthalate (PBAT), polybutylene succinate (PBS), polybutylene succinate-co-adipate (PBSA), polyhydroxyalkanoates (PHA), polybutylene succinate (PBS), polybutylene adipate terephthalate (PBAT), polycaprolactone (PCL), etc. The use of such alternative materials has increased over the years. Several studies have demonstrated the advantages of the use of biopolymers over the use of standard synthetic polymers [6,9,10,11]. However, considering the limitations of biopolymers in terms of mechanical, chemical, and environmental resistance, the use of biomaterials may be restricted to only a few applications; most commonly mulching. Due to their biodegradability features, after the cultivation period, biodegradable mulch films are typically left on the fields. Some studies on biodegradable mulch plastics indicate that the biodegradation and decomposition processes of these materials might be slower than expected due to the transportation of the residues with water and wind [12,13,14,15,16,17]. Although widely used in agriculture, there is still a lack of knowledge on the short- and long-term effects of biodegradable polymers on soil properties, namely on their interactions and effects on the soil microbial communities [12,13,14,15,16,17]. Further knowledge and adequate life cycle assessments, addressing a direct comparison between the influence of fossil-based and biopolymers on soil properties (carbon, toxicity, organic matter, and soil microbiome) and other life cycle-relevant aspects, is essential to provide guidance to regulators, policy makers, and farmers on the most adequate solutions for ensuring agriculture efficiency and productivity while maintaining and improving the safety and sustainability of agroecosystems.

### 2.2. Plastic Volumes in Agriculture

The FAO estimates that 12.5 million tons of plastic products are used worldwide in agricultural production annually, with films accounting for most of this quantity (approximately 60%). China is reported as the largest user of agricultural plastic, accounting for approximately six million tons annually [6].

**Figure 1:**
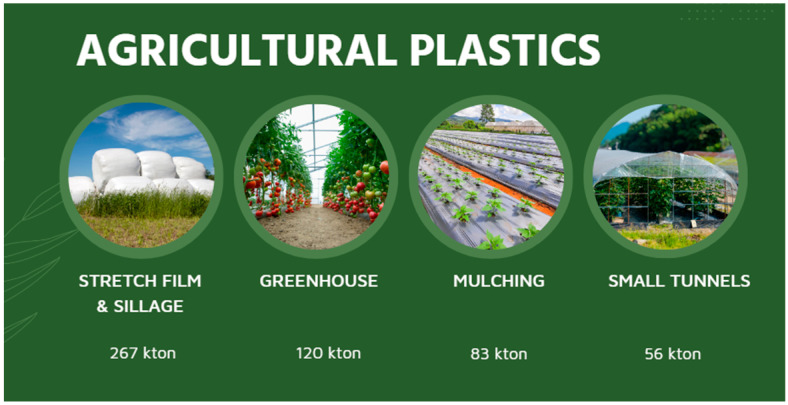
Main agricultural plastic applications in Europe in 2019. Source: adapted from reference [3].

Films used for mulching, silage production, and greenhouses constitute 50% of the total annual agricultural plastic volume. The remaining applications include irrigation tape, bale nets, plastic containers for the transportation of goods, and for phytopharmaceutical products [6]. The global figures available for agricultural films alone indicate an increase from 2018 to 2030 across all areas of the globe, with the highest increase expected for Asia (approximately 66% increase). Data from 2019 indicate that the European plastic sales volume associated with the agriculture sector was approximately 700,000 tons [3]. This constitutes approximately 3 to 4% of the total amount of plastics produced in Europe. Of these, 45% of the plastic was sold for vegetable production, and the remaining 52% was used for livestock applications. The APE Europe Figures from 2019 indicate that 41% of the overall plastic used in agriculture (approximately 1.73 million tons) was utilized for non-packaging purposes (0.71 million tons) [3]. Most of the plastic used in crop production is used in applications such as mulch films, greenhouse films, irrigation, and tunnels, whereas livestock plastics are typically associated with silage and stretch films, bale nets, and twine [3]. The primary use of plastics in agriculture is allocated to agricultural films; a total of approximately 650 thousand tons. Agricultural film applications include greenhouse films, small tunnel applications, mulch applications, twine, and silage films. Stretch film, greenhouse films, silage, and mulch film emerge as the dominant application segments (approximately 65%), followed by twine, small tunnels, and bale net applications (approximately 20%). The approximate amounts of some of the main applications are depicted in Figure 1.

**Figure 2:**
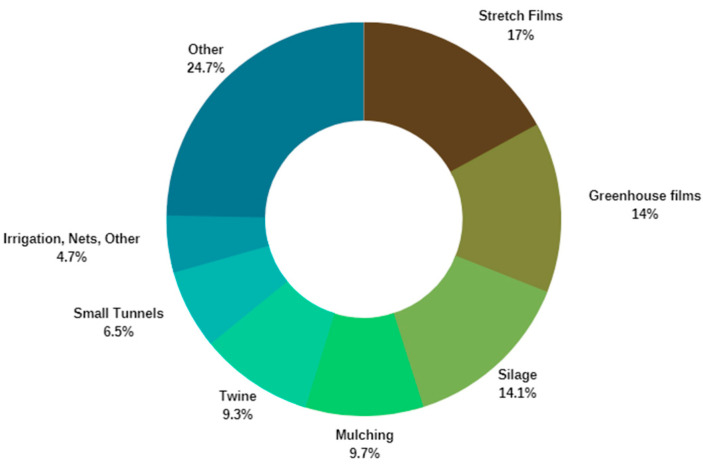
Percentual distribution of application segments for agricultural plastic (2019 figures). Source: adapted from reference [3].

As seen in Figure 2, the remaining agricultural plastic was used on irrigation, nets, packaging, and transport related items [2,3].

Reports indicate that most of these plastics are not properly collected, and that recycling and valorization of these residues is very limited [6]. This is mostly due to a lack of adequate collection schemes and policies specifically devoted to agricultural plastic waste. The exact figures on the amounts of agricultural plastic waste leaking into the environment are still not fully known. This is mainly because local collection and recycling/disposal infrastructures, as well as legislation, varies from country to country or is yet to be developed [6]. However, at a European level, considering the amount of plastics converted, consumed, plastic waste collected, and average plastic lifespan, one might roughly estimate the possible amount of agricultural plastic leaking to the environment. This topic will be discussed further in Section 5—Plastic production, Product conversion, and Waste Collection.

## 3. Product Development: From Feedstock to Final Applications

Agricultural films are structurally simpler than films used for standard packaging, as their production does not require the addition of additional layers made from other polymers or other materials to provide oxygen or moisture barriers. From this point of view, there is little space for design for recycling activities. The materials commonly used in these applications are standard, virgin grades, which are also used in other higher-end applications [18,19] and are sometimes precisely designed for use in agriculture applications [20]. It is worth noting that films made from recycled polymers are also becoming increasingly common, using materials made of blends of LLDPE and LDPE like the Circulen or NAV families [18,21]. As agricultural films are mostly made of one single material, they are inherently advantageous in view of the common recycling processes available [22]. Significant research has focused on the recycling of mulch films; however, there are also relevant studies covering bale wrap films [23].

An important aspect of polymer design and product conversion involves the introduction of measures that can lead to lifespan increases. This can be accomplished, for example, through the introduction of specific additives or through the optimization of, e.g., film thickness. Specifically, improving the long-term behavior of film materials can be achieved by tailoring anti-UV additives. This approach needs to be properly considered, as adding more substances to the original polymeric material might have negative consequences in terms of soil contamination and recyclability. Notably, the use of natural waste cornhusk can provide an environmentally friendly solution [24]. In view of the mechanical recycling of agricultural films, a particularly delicate subject is non-intentionally added substances (NIAS). Several studies have highlighted the absorption of pesticides into agricultural films, with no degradation of such pesticides once they are absorbed, as well as risks of contamination of the soil, water, and ecosystems placed in contact with these substances [25].

Downgauging, i.e., reducing film thickness, is an attractive measure, as it can reduce costs and the amount of plastic used within mulch films. However, the application of mulch films with higher thicknesses has been presented in the literature as a possible approach to increase the lifespan of mulch films and to simultaneously ease the process of removal of these films at their end-of-life stage. The use of mulch films with higher thicknesses enhances the retrieval rate after the end-of-life stage and increases their overall lifespan. Briassoulis et al. [26] showed that a proportional decrease in recycling output was obtained by decreasing film thicknesses from 40 microns to 20 microns. Incidentally, thicker films were shown to perform better in terms of their benefit to the crops. Fields where thicker PE mulching films were applied had the highest grain yield, as well as a lower release of phthalic acid esters (PAEs) into soil compared to biodegradable films [27]. However, the choice of the mulching film thickness is, other than adhering to the minimum standards fixed by regulatory bodies, largely left to farmers, who are, in the study from Li et al. in rural China, more influenced (44%) by the cost than by the performance for planting (23%) [28].

As an alternative to the perceived waste of energy and resources compared to their direct burning on the fields, studies on the possibility of recycling agricultural films started early [29]. Shredding and washing were identified as the critical steps for recycling, linked to the high stiffness of the films and the contamination with soil and chemicals. These contaminants can be as high as 40% wt. The effects of this high content of soil and contaminants on the mechanical recycling of these materials should be properly considered; for example, during the shredding step of films. Avoiding the presence of water during this process might be essential to avoid the development of an abrasive mud, which would lead to higher maintenance costs for parts of the mechanical recycling systems in the long-term, e.g., the blades.

**Figure 3:**
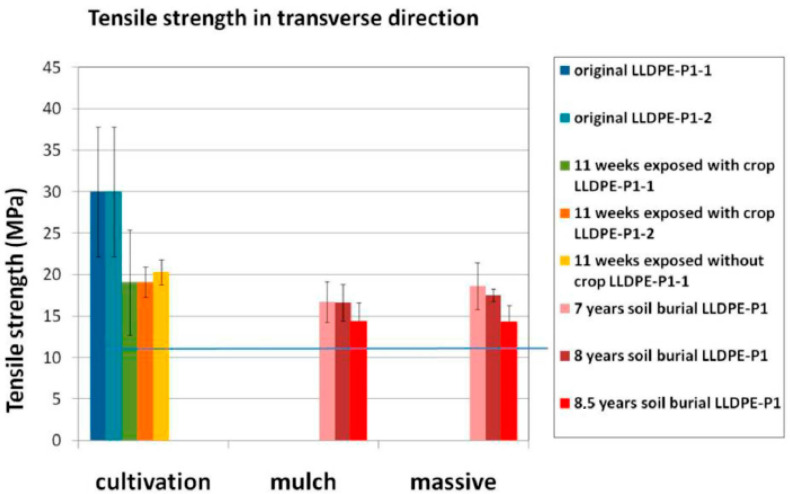
Changes in tensile strength with time and application. Reproduced with permission from ref. [30].

The characteristics of agricultural films, in particular with respect to their cleanliness from soil and contamination from, e.g., pesticides, make them suited mostly for mechanical recycling. From this point of view, the properties of the recycled materials are critically linked to the properties of the feedstock; therefore, the aging of these films has been carefully considered. Several studies [28,30,31] have reported on how the mechanical properties change with use, sometimes in studies as long as 8.5 years, as shown in Figure 3. In particular, the decrease in the elongation at break, i.e., embrittlement, and of tensile strength are often discussed, as shown in Figure 3.

**Figure 4:**
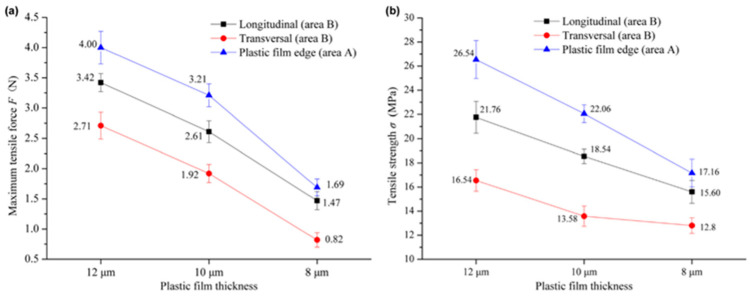
Dependence of mechanical properties on the film thickness. Note the area A, covered by soil, with improved properties. (**a**) Maximum tensile force as a function of plastic film thickness, (**b**) Tensile strength as a function of plastic film thickness. Reproduced with permission from Ref. [31].

Interestingly, it was also shown in ref. [31] and reported in Figure 4 that the decrease in the mechanical properties was less severe for the parts of the films covered by soil, thereby adding complexity to the subject. La Mantia [32] analyzed films for greenhouses, reaching the conclusion that for these materials, the limited worsening of processability and mechanical properties caused by the heavy stabilization packages used in the virgin materials allows for the possibility of recycling these films for the same use, i.e., in a closed-loop, when care is taken in the processing and re-additivation steps are considered. Picuno et al. also obtained good-quality recycled materials from greenhouse and low tunnel films by blending them with their HDPE film coverings, noticeably without additional additivation [33].

Reusing films is also a smart aspect of recycling. Building on the deterioration of the optical properties of used mulching films, Avissar et al. showed that the decreased reflectance of used mulching films caused more effective heating of the soil [34]. Alternative—albeit less environmentally friendly—strategies are also reported, like reusing the LDPE from agricultural films in multilayer film forms for the same purpose, coupled with a highly UV stabilized outer layer, as well as with the addition of EPDM to improve the processability of the degraded LDPE [35].

The addition of agricultural scraps to biopolymeric plastic matrices has been presented as an alternative to reduce plastic usage, thus decreasing costs, promoting an eco-friendlier process, and targeting a more circular economy approach. In addition to the advantage in terms of lower usage of plastic, this approach has shown to accelerate matrix biodegradability while enhancing mechanical performance [36,37]. This is a very promising approach to consider for the production of more sustainable plastic for agricultural applications.

The design of polymer and plastic products to be used within agriculture applications should always consider enhancements in the lifespan, reusability, and recyclability of the product. Appropriate designs should account for the entire lifespan of a product. Furthermore, and whenever possible, polymer development should focus on the use of mono-materials, which are easier to recycle and to valorize, after their end-of-life stage. For example, agricultural films should preferably comprise monolayers instead of multilayers. This approach is in alignment with Article 9(5) from Directive 94/62/EC, within which the European Commission addresses the need for a full life cycle assessment for products, addressing, in particular, waste prevention and design for circularity. The application of the Design for Recycling principles to products to be used in agriculture applications will facilitate the use of agricultural plastic waste as additional feedstock for mechanical recycling.

## 4. Public Policies, Instruments, and Governance Mechanisms for Plastic Waste

The initial steps towards the development of public policies, tools, and governance mechanisms that support initiatives for an overall improvement in plastic management across its full lifecycle were outlined in the 2015’s “European Commission Action Plan for a Circular Economy” [38]. The “European Strategy for Plastics in a circular economy”, proposed in 2018, has defined and integrated measures for plastics to be applied from cradle to grave and has set the framework for the development of adequate approaches within the full product lifecycle. This included polymer design, plastic manufacturing, product use, waste management, and waste recycling. Under this strategy, important measures were established, such as: (i) the restriction of use of microplastics, (ii) the reduction/banning of single-use plastic in the EU, and (iii) the assurance that all EU plastic packaging will be recyclable by 2030. Another important directive setting the framework for adequate development of public policies is the 2019/904 European Directive of 5 June 2019 [39]. This directive established goals and specific directions, focusing on reducing the impact of certain plastic products on the environment. Some of the principles outlined included the promotion of circular approaches, aiming at: (i) the development of more sustainable and non-toxic reusable products, (ii) the replacement of single-use products, and (iii) an overall reduction in the quantity of waste being generated. The directive focused mainly on the development of measures to prevent and reduce marine littering from plastics, with very limited reference to soil contamination and its risks for food security, water, and ecosystems. It referred to the need for developing proper waste management systems as a means of preventing all litter, complementing the regulatory measures that were already available within policy instruments related with waste prevention and waste management, such as Directives 2008/98/EC, 2000/60/EC, 2008/56/EC, and Council Regulation (EC) No 1224/2009 (10) [40,41,42,43].

The definition of the specific recycling targets for plastic packaging waste given in the European Parliament and Council Directive 94/62/EC (11) is another global measure set within the European Strategy for Plastics. This will ensure that, by 2030, all plastic packaging placed on the Union market is re-usable or easily recycled [44]. These measures are very much focused on the improper management of plastic waste used for packaging and its effects on marine pollution. The reference to agricultural plastic waste is very limited. This focused approach and lack of specific measures towards agricultural plastic waste might be due to the lower contribution of agricultural plastic waste (between 3 and 4%) in comparison with the packaging industry, which represents approximately 59% of the overall total plastic consumption.

Basic rules, policies, and regulatory mechanisms linked with plastic waste available in Europe have been for years based on the “polluter pays” principle, placing the single responsibility of polymer waste onto the ones that produce the waste. This approach considers that whoever causes waste should pay for its reuse, recovery, or disposal, leaving polymer producers, polymer converters, and other key actors out of the responsibility loop.

Global-, European-, and national-level public policies of different types have been developed to tackle and address the need for an improved circularity and recyclability of plastics. These policies are of different types and include direct regulations, economic instruments, and voluntary systems. The implementation of specific regulations for plastic waste management, separation, classification; the prohibition and limitation (in quantity) of the use of a given type of plastic for a specific application; and the prohibition of the irresponsible handling of plastic waste (e.g., burning, landfilling) are examples of direct regulation policies. Instruments aimed at improved stewardship and waste management of plastics, such as the “extended producer responsibility” (EPR), are another example of direct regulations. Direct regulations such as the ones defined above are designed to create obligatory standards and rules that need to be followed by key players. Fulfillment of these regulations is subject to control, and non-compliance leads to the implementation of a set of “punishment” measures.

Economic instruments are a type of policy instrument that typically support key stakeholders, encouraging them to follow the standards and rules defined within direct regulations. Examples of economic instruments include financial incentives/subsidies granted by governments for private entities to support the development of the more sustainable use of plastics and the development and implementation of systems, technologies and practices, leading to improvements in waste management and recycling. Economic instruments can include specific loans, grants, subsidies, feed-in tariffs, tax incentives, premiums, and general R&D funds (national, European, or other). Cash for return incentives constitutes another type of economic instrument. One example is the practice of collection of used plastic waste in exchange for a fee (e.g., applied for plastic bottles). In some countries, there are additional so-called tax break incentives, whereby a lower tax rate is applied in exchange for proven sustainable plastic waste stewardship. The application of fees and taxes are additional examples of economic instruments that can be used to penalize irresponsible plastic waste management practices (e.g., use of single-use plastics, lack of compliance with specific quotas for recycling, etc.). These can include fees, taxes, etc.

Finally, voluntary approaches typically enable the development of good codes of practice and positive changes in behavior. Examples include voluntary programs for plastic waste collection, sustainability certification schemes, and labeling (e.g., content of recycled plastic used within a product). The development of platforms for info sharing and communication, capacity building, knowledge build-up, and awareness initiatives also constitute examples of voluntary instruments.

Despite the confusion or apparent absence of binding legislation, farmers are the main actors in a genuine transition to a sustainable agri-food system, whether this is imposed by legislation, which will come sooner or later, or by the conscious practice of each actor in the sector. A first approach could be, for example, the gradual adoption of measures enabling the agricultural sector to comply with the International Voluntary Guidelines and Standards defined in the Codex Alimentarius developed by the FAO (Food and Agriculture Organization of the United Nations) and the WHO (World Health Organization), which is constantly updated (the latest version was recently adopted in Rome; 31 August 2023; ISBN 978-92-5-137755-0) [45].

Most of the existing mechanisms, policies, and legislation related to plastic and circularity do not address agricultural plastics in particular, being mostly focused on other type of plastics (e.g., packaging). These policies, conventions, and agreements are typically defined very broadly, failing to address specific aspects of agricultural plastic lifecycle, complexity, and broadness of applications, as well as difficulties associated with high contamination, low volumes, decentralized collection schemes, etc. Apart from some exceptions, in most European countries, there is no specific legislation/mandatory policies regulating the collection and recycling of agricultural plastic waste. The most regulated type of agricultural plastic waste is the one linked with the storage of chemicals, e.g., fertilizers, pesticides, and phytopharmaceutical products. In Europe, there is legislation and specific policies, providing guidance and clear rules on the management of plastic waste used for the storage of chemicals applied in agriculture. In Brazil, there are legally binding schemes which mandate pesticide producers, distributors, and end-users to collect plastic containers used for pesticides and other phytopharmaceutical products (inpEV Brazil Law nº 9.974/00) [46].

For the case of agricultural films specifically, to the best of our knowledge, except for Korea, Brazil, Ireland, and Norway, there are no mandatory schemes/policies obliging farmers to collect and recycle end-of-life agricultural films. Unfortunately, and despite the existence of mandatory legislation in Ireland and Norway obliging the collection and recycling of agricultural films, the data indicate that the collection rates for agriculture plastic waste in these countries is still only 70 and 84%, respectively. In some countries, there is specific legislation that indirectly supports easier removal/dismantling of agricultural films at end-of-life. This is the case in China, which developed, for example, specific legislation (standards GB 13735-2017 [47]) that establishes a minimum thickness for non-biodegradable mulch films.

In terms of the prohibition of plastics, an example of legislation at the European level is the ban on the use of non-biodegradable coating polymers in fertilizers due to their association with high levels of pollution and lack of circularity. This legislation is planned to be implemented by 2026 and was defined in 2019 (Regulation EU-2019/1009) [48].

Other examples of policies which indirectly promote an increase in the collection and recycling of plastic waste, and more specifically agricultural plastic waste, are taxation schemes applied to landfills, such as the ones used in the United Kingdom, Ireland, and other Northern European countries [49]. The imposition of landfill taxes, defined within the EU Waste Directives, has led to an increase in recycling rates. Within Europe, different approaches have been applied, which include the application of landfill taxes or landfill bans [49].

New policies associated with agricultural plastic waste should broaden the responsibility from users to additional parties involved on the plastic value chain (producers, retailers, and consumers). This means that the responsibility and costs associated with the collection, recovery, and valorization of plastic waste shall be covered, specifically for agricultural waste, by both the end users (farmers) and polymer manufacturers (film, pipe, and other producers). This approach constitutes a change in the paradigm for agricultural plastic waste management and places the liability on both users and polymer manufacturers. An important aspect to consider is the relevance of the involvement and the responsibility of polymer producers. As discussed in Section 3, an important input is the one from polymer producers, as material development and proper polymer design can be used to increase the lifespan of agricultural plastics. The need for more resistant plastics for agriculture applications extends the overall liability/responsibility for the appropriate use of agricultural plastics to polymer producers, in addition to polymer manufacturers and end users (farmers).

## 5. Plastic Conversion, Product Consumption, and Waste Collection

**Figure 5:**
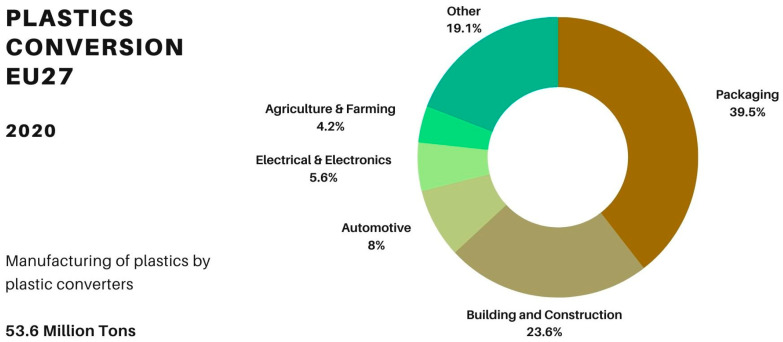
European figures for global plastic conversion (2020 figures). Source: adapted from references [3,51,52,53].

**Figure 6:**
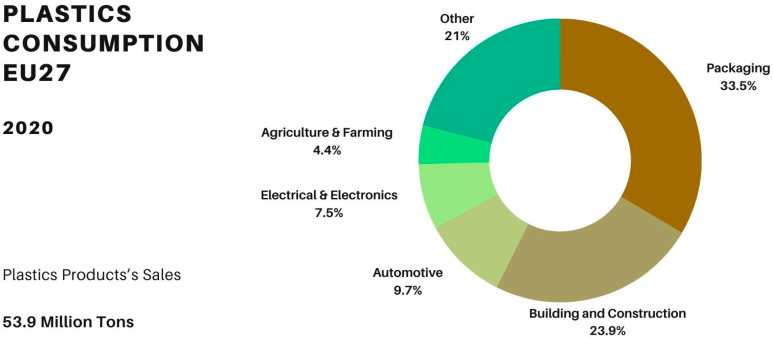
European figures for global plastic consumption (2020 figures). Source: adapted from references [51,52,53].

European statistics from 2020 indicate that the amount of plastic converted into new products and the consumption figures of products containing plastic as a component are very similar: 53.6 and 53.9 million tons, respectively (Figure 5 and Figure 6) [NO_PRINTED_FORM] [50,51,52,53]. The market associated with the highest production and consumption of plastics is the packaging industry, ranging from 30 to 40% of the total plastic volume (Figure 5 and Figure 6). Plastic applications and products linked with agriculture and farming constitute approximately 4% of the overall volume of plastic products produced and consumed (Figure 5 and Figure 6).

Considering the European statistical data only, the figures from 2020 indicate that the overall amount of post-consumer plastics collected was approximately 29.5 million tons [51,52,53]. Of this amount, 61% was linked with packaging products, 12% was for building, construction, electronics, and electric products, and 5% was used in agriculture products [51,52,53]. The lower volumes of plastic waste collected, in comparison with the production and sales volumes of plastic-based products, can be linked not only with the life cycle of the products (some products have a longer lifetime than others), but also with the available collection/recycling programs that are specifically developed for the different “classes” of plastic waste.

The post-consumer plastic waste collected figures indicate that the packaging waste collection systems are by far the most developed ones and constituted so far, with the highest percentage of overall plastic waste collected.

The amount of European post-consumer plastic waste sent to recycling reached 35% in 2020. The countries that performed best in terms of post-consumer plastic waste were the Netherlands, Norway, Spain, and Germany, with recycling rates exceeding 40%. Therefore, there is some space for improvement.

Programs and specific operating collection schemes for agricultural plastic waste are widely used in the USA, Canada, and some European countries. These programs offer different approaches and models, ensuring the collection of distinct types of agricultural plastics (containers, films, irrigation pipes, etc.). In most cases, these programs involve direct cooperation between actors from the overall value chain of agricultural plastics, enabling a full scheme that involves not only the collection of plastics but also the valorization step of these materials, e.g., through mechanical recycling and the production of new plastic products for different applications, including agriculture, gardening, and construction. These collection scheme programs consist mainly of voluntary systems which combine the efforts of farmers, recycling companies, and polymer converters.

**Figure 7:**
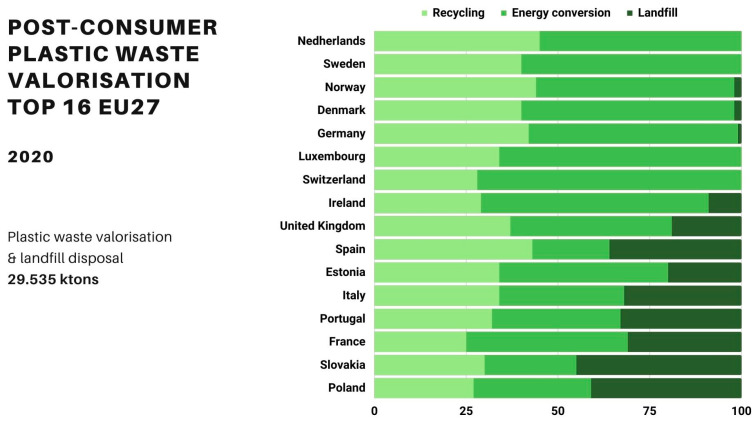
European figures for post-consumer plastic waste valorization and landfill disposal (2020 figures). Source: adapted from references [3,51,52,53].

The successful implementation and effectiveness of agriculture recycling programs has been higher in countries with specific legislation that addresses and considers the requirements and challenges associated with this specific type of waste. In Europe, only Sweden, Germany, Norway, Switzerland, Ireland, and France possess a proper/regulated collection system for agricultural plastic waste. Except for France, these are in fact in the list of the European countries for which higher recycling rates have been achieved for these and other types of post-consumer wastes (Figure 7).

**Figure 8:**
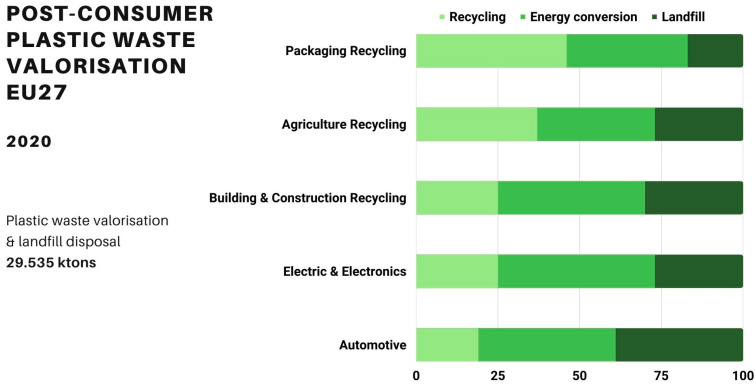
European figures for global plastics conversion (2020 figures). Source: adapted from references [3,51,52,53].

Statistical European data indicate that the development of adequate collection schemes, supported by appropriate public policies, has led to improvements in the treatment and valorization of agricultural plastic waste. Figures from 2020 from Plastics Europe indicate that of the overall 1,481,000 tons of agriculture, farming, and gardening waste, 37% was recycled, 36% was used for energy valorization, and 27% was landfilled [52]. Agriculture plastic waste has the second-largest position in terms of recycling rates, immediately after plastic packaging (Figure 8). The overall volumes of packaging recycling recovered, in addition to the amounts ending up in landfills, was 17,946 tons.

Since 2018, agriculture plastic waste has experienced, together with packaging, the largest increase in recycling shares: an approximately 4% increase between 2018 and 2020. This reflects the importance of the development of adequate and focused collection schemes and their positive effects in terms of recycling rates.

The development of new advanced technologies for washing, shredding, and mechanical recycling of agricultural plastic waste and the availability of other alternative valorization schemes (e.g., energy recovery, chemical recycling, other) will, in coming years, further lead to increases in both the recycling and energy valorization of agricultural plastic waste. These approaches are further developed in Section 6 and Section 7.

Nevertheless, and despite recent technological developments, there is still further improvement required to further decrease the amounts of agricultural plastic waste that are sent to landfills. Solutions and approaches to target this objective will be outlined in the next sections of this publication.

But what do we know exactly about existing collection programs for agriculture plastic waste? So far, some of the most relevant organizations being developed in Europe, aimed at the recycling and valorization of plastic wastes originating in the agriculture industry, include the Agricultural Plastics Environment Group (APE) [3], the consortium ERDE (Crop Plastics Recycling Germany) [54], the Finnish-based Maatalousmuovien Kierrätys Oy organization [55], the Scottish Zero Waste program [56], the Spanish-based association MAPLA [57], etc. Their common approach consists of creating consortia covering the full value chain of agricultural plastic waste, which includes polymer producers, polymer manufacturers, recyclers, and retailers. These associations aim to increase the collection, recovery, and valorization of agricultural plastic wastes. So far, the final applications for the agricultural recyclates collected through these organizations have been mainly focused on garden furniture, fences, plastic bags, grain bags, agriculture recycling films, and other materials used for construction.

The main challenge of all these initiatives is to create sustainable business models and an overall profit for those involved in the collection and valorization of these materials. In most of the cases, the recycled product has an inferior quality compared to the original virgin product; therefore, only permitting the use of the recycled polymers in lower-value applications, as expected, has an associated low profit and margin (down cycling). Improvements in the applied separation techniques, immediate collection and waste segregation by farmers, adequate training on best separation methodologies, the development and the application of modern cleaning, shredding, and mechanical recycling technologies is thus essential. Significant interest and research has been directed towards the use of mechanical recycling for the development of recyclates with improved organoleptic, color, and mechanical performance. Such materials were achieved through the use of advanced separation, washing, and color segregation technologies, leading to materials with improved properties, approaching the ones obtained using virgin materials [18,21,58,59,60]. This was, however, mainly focused on recyclates produced from packaging post-consumer waste and should be further developed to target an equally challenging class of plastic waste: agricultural plastics.

Another significant challenge is the fact that these collection schemes are mainly voluntary based, with no or very limited associated political instruments and incentives that are able to motivate farmers and recyclers to further develop collection and valorization schemes. In any case, these are valuable initiatives, which should be used as models and serve as the basis for further development. So far, since its inception, the APE has managed to collect and recover approximately 38 476 tons of agricultural plastic waste. The plastics which have been collected within the Maatalousmuovien Kierrätys Oy project so far have been used for the production of new agricultural plastic solutions, such as the RaniWrap Eco bale wrap. A similar approach has been used in the Zero Waste Scotland program, which has resulted in the development of recycled plastic bags known as the Green Sack^®^. As for the Spanish-based association MAPLA (Medio Ambiente, Agricultura y Plásticos), since its creation, the free cost and voluntary collection of agricultural plastic waste at specific assigned collection points or directly at their premises (farms), has resulted into the valorization of 100 thousand tons of agricultural plastic waste.

As previously pointed out, most European countries do not have collection and valorization schemes that are specifically devoted to addressing the challenges of agricultural plastic waste. In these countries, the focus so far has been on the development of collection and recycling programs for packaging plastic waste. An example of this is Portugal. Existing Portuguese collection schemes are limited to the management of plastic packaging used for chemical and phytopharmaceutical products, biocides, and seeds. Valorfito is an integrated system for the management of this type of plastic, which was built to support farmers in complying with existing legislation that obliges the collection and recycling of packaging used for agro-chemicals and seeds. In line with Portuguese policies (specifically Decreto-Lei n.º 178/2006 de 5 de Setembro, com alterações do Decreto-Lei n.º 73/2011, de 17 de Junho), the Portuguese Regional Direction for Agriculture and Fishery establishes the liability of the final users (farmers) to the management, costs, and transport of agricultural plastics and prohibits practices such as burning, abandonment in the field, and burying of these residues. Recycling, energetic valorization, and other practices, such as landfill disposal, are mentioned as possible alternatives for plastic waste management. Guidelines/recommendations for final users are given through the Portuguese Regional Direction of Agriculture and Fishery webpage [61]. These measures have significantly reduced previous practices such as burning and landfilling, but they only solve the issue with chemical containers, leaving other agricultural plastics such as films and pipes out of the loop for collection and valorization. According to data from the Associação Portuguesa do Ambiente (APA), in 2019, plastic wastes originating from agriculture practices were received by different waste management organizations and urban waste management companies, and parts were exported to Slovenia, Spain, and Latvia. Data from 2021 showed that more than 40% of plastic waste produced in Portugal was still ending up in landfills. This is just an example that reflects that there is still space for improvement.

Taking into consideration the type of collection initiatives for plastic waste in Portugal, current legislation, and the percentage of generated plastic waste that still ends up in landfills (40%), one can state that the development of adequate collection and plastic waste management systems in Portugal still has significant potential for improvement, ensuring a more sustainable use of plastics in agriculture and their appropriate valorization after their end of life. In a recent publication, researchers pointed out that there is still significant potential for valorization of plastic waste in Portugal. Apart from standard mechanical recycling technologies and energy recovery, pyrolysis, gasification, and even hydrogen production are mentioned as possible alternatives to the currently practiced landfill disposal approaches [62]. Regardless of the initiatives and overall discussion on the improvement of European plastic waste management practices, the central focus of the approaches has still been mostly on plastic waste originating from packaging, with no or very limited focus on agricultural plastic waste.

In conclusion, and as clearly illustrated in Figure 7, many European countries, such as Portugal, Italy, Spain, Estonia, Poland, and even France, still have significant work to do in order to decrease the overall amounts of plastic waste sent to landfills.

## 6. Mechanical and Chemical Waste Recycling Approaches and Technologies for Plastic Waste Recycling

There are different methods for the recycling, valorization, and disposable of plastic waste, which include mechanical recycling, chemical, etc. [63]. The best approaches and processes for the recycling of agricultural plastic waste are mainly determined by the material composition, original type of application, water content, and level of contamination. The steps and processes required for the valorization of agricultural films used in greenhouses, mulch films or piping are therefore, different. In general, agricultural mulch films have medium to high contamination, consisting of soil, sand, small stones, additives, metals, and other chemical contaminants (such as residues from phytopharmaceutical products). Therefore, prior to shredding and the application of a given recycling process, these materials need to be properly decontaminated. Agriculture film recycling and shredding, in particular, are still very challenging, considering that in some cases, the residue percentage can be within the range of 40 to 50%.

Mechanical recycling of agricultural plastic waste has been one of the main valorization processes used. The latest advances in terms of waste separation and mechanical recycling technologies have made the achievement of highly pure and high-quality polymer materials possible, using either pure or combined with virgin resins to produce new products. The main developments in mechanical recycling technology have been associated with the development of advanced composition characterization systems (NIR, hyperspectral analysis, etc) applied for the segregation of materials upstream the mechanical recycling process [58]. This, combined with pre-treatment, conditioning units, advanced filtering units, degassing, and extruder profile combinations adequate for improved mixing, has led to products with improved properties [18,21,58,59,60,64,65,66,67]. The development of technologies enabling pure recyclates with very high purities (higher than 98%), improved organoleptic properties, and enhanced end-performance (color, mechanical performance, etc.), has resulted in a lower need to mix virgin materials with recyclates. This has led to an increase in “pure” recyclates with low levels of virgin material incorporation, serving as a boost in recyclability and an improvement in customer confidence of recyclate materials [59,60]. When considering agricultural plastics only and agricultural films, in particular, advanced mechanical recycling plants now possess state-of-the-art technology, enabling improved separation, segregation, decontamination, washing, and shredding, specifically adjusted to the special requirements of agricultural film recycling (high contamination, high moisture, high degradation) [64,65,66,67]. Features such as polymer composition, purity, level of contaminants, flow behavior, transparency, color, volatiles, and mechanical performance will define new possible applications for recycled agricultural plastic waste. Some examples include new agriculture film, bags for storage, garbage bags, furniture, fences, damp-proof membranes, etc. [64,65,66,67].

**Figure 9:**
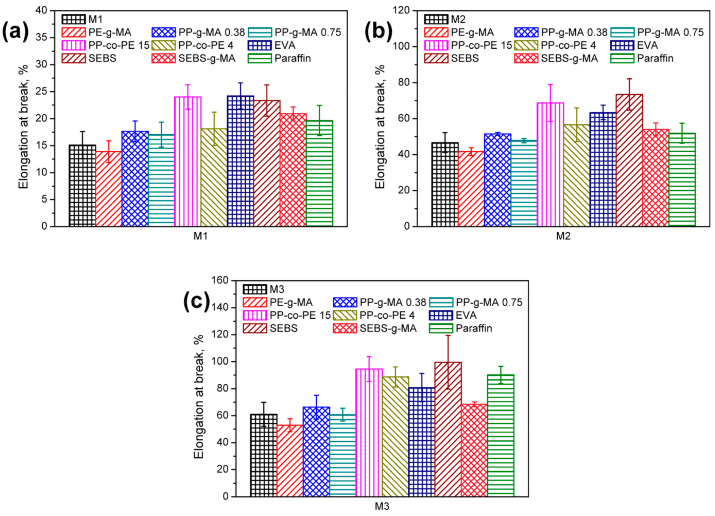
Improvements in the elongation at break of recycled polymers from agricultural films depending on the different compatibilizers used. (**a**) M1—waste material after one washing-drying step (**b**) M2—waste material after two cycles of washing-drying (**c**) M3—waste material after three cycles of washing-drying. Reprinted from ref. [32].

For materials characterized by a high level of contamination, improvements in material homogeneity and ductility might be achieved through the incorporation of a residual amount of standard compatibilizers, e.g., maleic anhydride-grafted polyolefins, SEBS block copolymers, EVA copolymers, or paraffin wax [68]. Figure 9 shows the detailed change in elongation at break from reference [65], depending on the compatibilizer used. In general, a relative increase of up to 64% was obtained for the elongation at break.

Coextrusion of blends of recycled and virgin materials originating from greenhouse covering has been investigated by La Mantia et al. [32]. The researchers found that the best resulting properties of the films were obtained using virgin and recycled material monolayer blends or coextruded blends in which the recycled material was placed between two layers of virgin polymers.

Chemical recycling is another technique for the valorization of plastic waste. This process is technically more complex than mechanical recycling. There are essentially two types of chemical recycling: chemical depolymerization (solvolysis) and thermal depolymerization. The latter can be achieved through pyrolysis (in the absence of air) and gasification (under controlled conditions) [63]. These processes target different polymer waste feedstocks. Depolymerization is mostly used for highly pure polymer waste streams (monostreams with 98% purity) consisting of polyesters, polyethylene therephthalate (PET), polyamides (PA), polyurethanes (PU), etc. Pyrolysis is typically applied to mixed streams consisting of LDPE, HDPE, PP, PS, PET, nylon and PVC pyrolysis. Thus, this technology remains more challenging. Mixed polymer streams of higher complexity are typically better suited to gasification based chemical recycling [69]. Chemical recycling operational costs are still very high in comparison with mechanical recycling approaches. High yields and lower costs are typically obtained via the use of very pure feedstocks, with low levels of contaminants. This is, therefore, not the preferred approach for valorization of agricultural plastics, at least for now, considering existent processes and technology.

In conclusion, of all the above-mentioned processes, mechanical recycling appears to be the most promising in terms of technical feasibility, costs, and profitability. Significant improvements in terms of the separation and cleaning technologies used for segregation and valorization of agricultural films have resulted in materials with fewer contaminants (soil, chemical residues, etc.) and lower moisture levels. This, together with the relatively simple composition of agricultural plastics (typically less complex than packaging, automotive, and electric/electronic applications), brings the recycling and valorization of agricultural plastic materials via mechanical recycling potential to another level. Current technological developments, which combine efficient preconditioning and drying units, melt filtration, homogenization, and degassing in a single step, make the mechanical recycling and processing of complex materials with heavy contamination and high moisture technically and economically feasible.

## 7. Other Agricultural Plastics Waste Valorization

Other ways of valorizing plastic waste arising from agriculture involve specific approaches or processes. Most of them come about when others, especially those that are not final steps, are no longer possible. These processes include those that allow plastic waste to be incorporated or even transformed into, for example, fashion pieces, building materials, or road and railway pavements [70,71,72,73]. This set of approaches is of the utmost importance, as can be seen when comparing these processes, often referred to as end processes, with other, very actual realities, which lack control over the total dumping of this plastic waste in the environment (where it accumulates both in the soil and in fresh and saltwater) or over its increase in landfills. This includes approaches where plastic waste is incorporated into products such as green roofs, bricks, garden furniture, stakes for various crops (e.g., vineyards, fruit orchards), footpaths (plastic decking and lumber), asphalt mixes, railway sleepers, and many others, like sneakers [70,71,72,73,74,75,76,77].

Another approach is to submit this plastic waste, which is often highly dirty (contaminated with soil, plant residues, some metals, etc.), to thermal processes, usually in a controlled atmosphere, in order to obtain other materials or value-added products. These include the production of adsorbent materials, gases with energy value, and biofuels [78,79,80]. In the case of adsorbents, it has been possible to obtain adsorbent carbon materials, prepared through physical and chemical activation processes, with potential applications in the removal of hazardous molecules and pesticides present in the aqueous phase [80,81]. 

The production of hydrogen for energy purposes has been another focus of academic and industrial research in recent years [78,82]. In addition, some authors have succeeded in producing energy and syngas (a gas mixture composed of varying amounts of carbon monoxide and hydrogen) from such wastes using thermochemical gasification processes and by exploiting the remaining fraction of solid by-products and converting them into adsorbent materials [81,82]. One of the most widely used forms of this type of conversion is the use of a closed system—an autoclave—where, at a low temperature range (usually less than 400 °C) and with the addition of water, it is possible to increase the yield of the plastic waste conversion process. This process, known as HTC (hydrothermal carbonization), is characterized by the production of liquid and solid products—coke and char, respectively—which can be further processed for their calorific value [83]. HTC treatment almost always involves the addition of other materials to the plastic waste to be converted, usually some biomass waste [84,85]. The continuous increase in the price of traditional fossil fuels has led to a strong demand for alternative liquid fuels [79,86,87] and to the development of processes not only at a large scale [88] but also at a local and regional scale [89]. This has led to the production of liquid fuels from agricultural plastic waste, which are mainly used as an alternative road fuel and, in some cases, as a fuel for heating systems.

It is important to note that many of these processes and approaches have advantages at local and regional levels, particularly in less-developed areas. They have the potential to generate civil society and farmer movements to collect this plastic waste, thereby making a decisive contribution to reducing the pollution of terrestrial and aquatic systems through the dispersal of this waste.

## 8. Microplastics, Soil Remediation, and Restoration Strategies

In a recent review, Tian et al. comprehensively described the main sources of microplastics entering agricultural soil, including agricultural practices using plastics (mulching, greenhouses, etc.), the use of plastic carriers for seeds and fertilizers containing plastic, irrigation, and atmospheric deposition [90]. Once in the soil, microplastics undergo photo-degradation and mechanical abrasion and may interact chemically with other species, namely fertilizers, pesticides, and heavy metals. Such interactions result in changes in the soil bulk and chemical properties, including pH, density, and porosity. Other soil features, such as the soil microbiome, which are strongly linked with soil health and productivity, may also be affected. In addition to the negative effects of microplastics on soil health and productivity, there are also concerns regarding their consequences to ecosystems and the exposure of humans through food and water chains. In a recent study, researchers investigated the nature of microbial biofilms—the so-called “plastisphere”—that colonize microplastic surfaces [91]. They found that this community, when attached to plastic that has come into contact with feces (such as those from organic manures, wastewater, or animal waste), as is often the case for agricultural plastics, might be associated with human pathogens. As the removal of microplastics from plant leaves (even through washing) is a challenge, there is the risk that pathogens remain attached during the harvesting and retail processes, increasing the potential for human pathogens to enter the food chain. In addition, the “plastisphere” can increase the survival and dissemination of human pathogens, facilitating their transfer to the rhizosphere, the roots, and the surface of leaves and fruits, either directly through irrigation or indirectly through splashing from the soil.

Researchers agree that further research on these areas is needed to understand and evaluate the short- and long-term effects of microplastics on soil, ecosystems, and humans. A more in-depth knowledge is required, including long-term monitoring of soil key properties, evaluation of the effects of the exposure of soil and ecosystems to microplastics, and a systematic investigation of the composition and main characteristics of microplastics found in agricultural soils [7,92,93].

The introduction of microplastics into the environment stems from various sources, including airborne deposition and the degradation of plastic materials in farming practices, especially plastic-containing mulch. This leads to elevated concentrations of micro(nano)plastics in agricultural soil, which can have far-reaching consequences.

Microplastics within agroecosystems can impact both fauna and flora, with potential implications for human health through trophic transfer, ingestion, and inhalation [94]. Furthermore, microplastics may hamper agricultural productivity by delaying germination, reducing transpiration rates, and inhibiting plant root growth, posing potential threats to food security [95]. These persistent microplastics necessitate a multifaceted approach to mitigate their impact and facilitate remediation. Strategic land management practices, such as reduced or no-till farming, can effectively limit the spread of microplastics, while the incorporation of organic matter or soil amendments like activated carbon and biochar can curtail their mobility [94].

Remediation strategies for microplastics encompass physical, chemical, and biological approaches. Physical and chemical techniques are typically faster, but they lack a biological dimension. In contrast, biological methods are noteworthy for their environmental sustainability [8,96]. Microbial degradation of plastics is influenced by various factors, including enzymatic processes, substrate concentrations, pH levels, oxidative stress, and temperature [94]. Microorganisms capable of utilizing plastics as a carbon source, such as bacteria, fungi, and actinomycetes, can partially or entirely break down plastics into biogas and biomass [8,94,97]. Ongoing research explores additional methods, such as enzyme and membrane technologies, nanoparticle technology, and metagenomics [98]. Nonetheless, challenges persist, particularly in identifying microorganisms capable of targeting a wide range of plastics and high molecular weight polymers [94,98].

In summary, the diverse array of techniques used for microplastic remediation calls for careful consideration of site-specific conditions and remediation timelines. Monitoring remains imperative to assess remediation effectiveness and potential long-term repercussions.

## 9. Discussion

According to United Nations projections, the world population is expected to rise to 9.8 billion by 2050 and 11.2 billion by 2100. This projected population growth will require a global increase in food supplies. According to FAO data, the assurance of food safety will require a 60% increase in overall food production. As a result, there will be a requirement for an expansion in the use of agricultural plastics and the need for more efficient and sustainable agriculture practices. Therefore, the 3% current share of agricultural plastic waste in total plastic consumption is also expected to increase.

A critical aspect for the assurance of food security is the availability of healthy and productive soil. Research indicates that several pressures, namely overuse, inadequate management, and environmental and climate change-related challenges, are a significant threat to soil health. In Europe, 60 to 70% of soils are already degraded, with a significant loss in terms of productivity and also a severe reduction in the capacity to provide ecological functions for various forms of life. Bearing in mind the finite nature of this resource, each and every action towards improvements in soil health and soil regeneration is welcome and urgently needed. The contamination of soil from plastics, resulting mostly from inadequate management of the plastic waste used in agriculture, is only one of the few considerable issues which need to be addressed. Tackling the technical, managerial, societal, and political issues related to agricultural plastic waste is very urgent, given the negative short- and long-term effects on soil, water, and ecosystems resulting from an inappropriate collection, disposal, and recovery of agricultural plastic waste.

An overview of existent solutions, together with a proposal for the introduction of a holistic approach for agricultural waste management combining the input of different actors and disciplines, will be the main focus of this discussion.

The main vectors/strategic areas which would need to be addressed to develop a holistic approach to tackle the issue of inefficient waste management and valorization of agricultural plastics include: (i) information gathering; (ii) legislation and public policy; (iii) research and development; (iv) business development; and (v) knowledge exchange, capacity building, and literacy improvement. By analyzing the requirements within each of the vectors above, one can state that the implementation of a robust system for agricultural plastic waste requires the acquisition of information at different levels and making this information readily available. Such information includes, e.g., data about agricultural plastic sale volumes; figures regarding the amounts of agricultural plastic waste sent for landfill disposal; the amount of agricultural plastic waste collected, recycled, and recovered; etc. Information regarding available collection schemes, locations for collection and storage of materials, and waste management companies and recyclers already working with agricultural plastic waste and/or with potential (technical, economical) is essential to start applying this waste feedstock. Knowledge on the potential alternative business models for agricultural plastic waste valorization (e.g., energy, activated carbon, etc.) aiming to increase profitability for this feedstock is, in addition, very important. The availability of this information in the form of open sources through online digital platforms and the assurance of transparency would be the ideal path to better control and monitor the progress of any implemented strategies at different points along the value chain.

The legislation and public policy vector needs to identify and analyze the following: (i) existing legislation, instruments, and relevant mechanisms relating to agricultural plastic waste; and (ii) technical and socio-economic barriers to the implementation of existing and future public policies and instruments. This information, together with other aspects derived from R&D and business development, will serve as the basis for the adaptation and implementation of existing/new public policies for agricultural plastic waste management and valorization.

The research and development vector is essential to enable the design of polymers for agricultural plastic applications that are more conducive to recycling, recovery, and reuse. R&D activities will also enable us to verify the technical and economic feasibility of the recycling and recovery/valorization approaches applied to agricultural plastic waste. Here, the key success factor is to ensure that R&D projects include activities covering the full value chain of the products, starting already at a polymer design level (through, e.g., the implementation of design for recycling strategies for agricultural plastics) and ending at the plastic waste level.

The development of economically feasible approaches for the improvement of agricultural plastic waste assumes that alternative/new business models should be established. This is dependent on the outcomes, synergies, an interconnections between R&D and the legislation and public policy vectors.

The knowledge exchange, capacity build-up, and literacy improvement vector is another area that should be targeted. The importance and relevance of the establishment of adequate waste management and valorization schemes for agricultural plastic waste, and the dissemination of this knowledge to key stakeholders, is very important. For example, illustrating the importance of the adequate management of plastic waste and highlighting its positive effects in areas such as soil health, water quality, and food security is essential to ensure the acceptance and compliance of the new management/valorization models at different societal levels. Knowledge of the best practices and technologies within the full value chain is relevant for all sectors/actors, namely the polymer industry, farmers, civil society, policy makers, waste management companies, recyclers, and other industries of relevance for development of valorization approaches and products for agricultural plastic waste. The knowledge exchange, capacity building, and literacy improvement vector also includes: (i) the development of institutional support for farmers and other parties involved in the waste management and valorization processes, (ii) the establishment of marketing and communication strategies targeting multiple actors (civil society, investors, farmers, etc.), and (iii) the creation and development of value chains associated with the valorization of agricultural plastic waste.

What are some exact examples of measures that should be taken to move forward, and what are the gaps that need to be filled? Of all the plastics used in agriculture and fishery-related areas, only agricultural plastics used in marine environment and plastics used for the storage of chemicals have international public policies and instruments regulating to their use, collection, and recycling [2]. For the remaining agricultural plastics, there are only a few scattered and very limited international instruments available, and very different approaches are being applied in different countries worldwide [1,2,6]. The “polluter pays” international environmental law principle is the basis for most of the voluntary-based systems available in some countries for the management of agricultural plastic waste [55,56,57,61,99,100,101]. These can serve as the basis for the application of adequate multi-actor cooperation models, but they cannot solve the multiple challenges that still exist in this area on their own. Further legal and/or policy action, together with adequate collection, recycling, and recovering schemes, are essential for the development of a technical and economically feasible model for agricultural plastic waste management and valorization.

The application of a 6R approach has been considered in many business areas as a way to improve sustainability within the full value chain of a product. The 6R approach is founded on various practices aimed at design improvements, a reduction of waste, recycling, reuse, and even the selection/refusal of products based on their sustainability and environmental footprint. Addressing the issue of agricultural plastics, in particular, implies the definition of a few measures which can ultimately lead to improvements in the sustainability of these materials and the consequent protection of soil, water, ecosystems, and their related services. A contextualization of the 6R approach measures within the frame of agricultural plastic waste is discussed below.

### 9.1. Reduce and Rethink

Reductions in agricultural plastics can be achieved through the development of new polymeric materials and/or, whenever possible, the application of more “nature-based” solutions. The use of biobased materials versus the use of fossil-based materials must be the subject of adequate/systematic studies. Each solution should be adjusted according to the requirements of the application and a validated long-term sustainability assessment. Adequate polymer design has been shown to be a potential solution to reduce the amount of polymer required for a given application (e.g., mulch films). Fine tuning of raw polymeric materials, together with adequate additives and polymer processing techniques, can result in improvements in mechanical resistance, which ultimately lead to increases in product life expectancies. Another parallel measure should be the establishment of adequate policies and governance mechanisms that are capable of regulating and, if needed, banning certain applications (e.g., establishing a minimum thickness for a film, defining a minimum mechanical resistance for a product, etc.). Such measures enable the use of fewer non-renewable resources and raw materials and lead to reductions in the amounts of energy and resources used for the production, transport, and application of products.

### 9.2. Recycle

Prior to recycling, adequate schemes for the collection of agricultural plastics must be implemented. Efficient and adequate collection schemes will enable the recyclability of agricultural plastics and their use, either in the form of new products for agriculture applications or in other areas of business (e.g., construction, furniture, energy, etc.) [62,71,74,84,85,102]. The collection and recyclability of agricultural plastics will only be viable through the implementation of measures that are capable of developing technical and economically viable business models. The approaches to be followed should be implemented in a way that balances the distribution of costs and benefits across the value chain. The development of a model for collection and recycling approaches should thus have an intersectoral, interdisciplinary nature involving multiple stakeholders from the full value chain, including polymer producers, polymer conversion companies, distributors, farmers, and recyclers. The implementation of robust and sustainable collection and appropriate recycling schemes needs to be accompanied by adequate public policies, instruments, and incentives. These should be legally mandated via the application of incentives, penalties, and obligations applicable across the entire value chain. Such policies must be accompanied by adequate control and management of sales records for new products as well as agricultural plastic waste records. This will make the evaluation of the effectiveness of the applied measures (collection, recycling) possible and serve as a supporting mechanism for the application of regulatory and compliance assurance measures (incentives, penalties, etc.) of the different public instruments in place.

The development of a sustainable and technically feasible collection system needs to be accompanied by adequate separation schemes/technologies and access to waste recycling infrastructure with a sufficient capacity. For example, segregation of products by farmers at the “source” during the collection processes can save separation costs and reduce contamination during the separation and recycling performed at waste management companies.

Barriers to the collection and recovery of agricultural plastic waste should be identified and minimized. One example is the development of methodologies and training to increase landowners’ literacy in terms of plastic separation and to improve the conditions and approaches for the collection and storage of waste. It is essential to promote both the collection and valorization of agricultural plastic waste through the development of new business models involving farmers, supported by both technical and economic feasibility studies.

The economic feasibility of agricultural waste collection and recovery might require the adjustment of existent EPR schemes, which are currently mainly focused on urban areas requirements. At least in some countries, EPR schemes will require adaptations to different type of users, including small-, medium-, and large-sized farms. Small farmers alone might not have a “critical” mass in terms of agricultural plastic waste volumes, which could render the introduction of collection and recycling schemes a severe financial burden. In these cases, “communal” collection and storage facilities might be necessary. This should be applied on top of the currently existent voluntary farmer and waste collection/re-processing led initiatives, such as the ones defined in Section 5 of this publication.

The application of the EOL approach for the management of agricultural plastic waste, which internalizes the costs required for recycling into the selling price of final agricultural plastic costs, could be a good solution to the financial burden of waste management in some agricultural plastics.

There are no exact figures on the percentage of collection rates that are actually recovered/recycled, since information related to the level of contamination and rejection rates from recycling facilities is not public knowledge. This information is essential to address possible needs in terms of the development of more adequate technologies/approaches for decontamination and recycling.

The absence in most of the countries of mandatory collection schemes, lack of infrastructure with capacity and technical adequacy to recycle contaminated agricultural plastic waste products, and nonexistence of incentives and penalties to motivate/enforce the collection of agricultural plastic waste, are some of the most important barriers for the development of robust/adequate Extended Producer Responsibility (EPR) schemes agricultural plastic waste.

### 9.3. Reuse

The reuse of agricultural plastics as such is difficult without reprocessing the materials. This is mostly due to the known contamination of films and other plastic products used in agriculture. Reuse of agricultural plastic products might require a rethinking in terms of polymer and product design, enabling the partial or complete reuse of some products with minimal reprocessing. The reuse of agricultural plastics is, however, not as straightforward as the recycling itself.

### 9.4. Refuse

The adoption of given consumer measures and behaviors, from, e.g., farmers themselves and consumers, can be used as a regulator for agricultural plastic use and agricultural plastic waste management. The refusal of a consumer to buy a product that is known to be produced following non-environmentally friendly and non-sustainable practices and the refusal to invest in companies that are not environmentally conscious (practiced, e.g., by banks and investors) are just some examples of “refusal” practices that could be applied to agricultural plastics. These practices are beneficial only when adequate incentives, certifications, and other benefits are applied to products being produced following more environmentally friendly practices (e.g., products retrieved from companies with robust collection and valorization schemes in place for agricultural plastic waste).

### 9.5. Repair

The repair principle might be suitable only for some types of agricultural plastics. In this case, polymer design is essential and requires solutions such as self-healing, which could be applied to some extent in some products, expanding their useful working lifetime.

One last important step towards the successful implementation of collection and valorization schemes for agricultural plastic waste is the acceptance and buy-in from farmers themselves, who will be ultimately the main driver for the overall process. Several socio-economic studies have been undertaken to evaluate the overall acceptance and attitude of farmers with regard to agricultural plastic waste management and the possible negative impacts of plastic waste for the environment [103,104,105]. In one of the studies, it was concluded that most of the interviewed farmers had good perceptions concerning the need for developing more sustainable models for the management of plastic waste. However, these farmers associated this need with a logistical and financial burden. In general, the farmers seemed to be more aware of the possible negative effects of plastic pollution into aquatic systems, but most of them tended to be unaware of the impacts of plastic contamination on soils, ecosystems, and productivity. These two facts demonstrate the urgency of increasing the literacy of farmers and civil society in general regarding the environmental, social, and economic importance of adequate agricultural plastic waste management and valorization.

**Figure 10:**
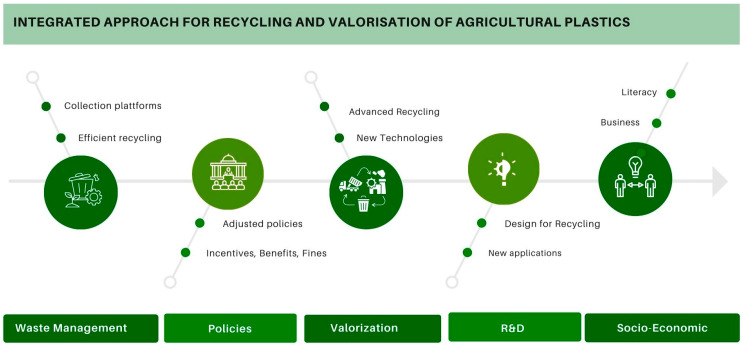
Integrated approach for recycling and valorization of agricultural plastics.

In conclusion, only a combination of the different remediation approaches for soil contaminated by plastic waste and other contaminants, together with adequate management and valorization of agricultural plastic waste, can lead to healthier and more productive soils (Figure 10). The establishment of strategic land management and emerging technologies offer promising avenues to address this critical environmental challenge. Monitoring remains imperative to assess remediation effectiveness and potential long-term repercussions.

## Figures and Tables

**Table 1:** Examples of typical materials and applications of plastics within agriculture.

Material	Main Applications
LDPE	Greenhouses, mulching, silage
HDPE	Chemical containers, shade, protective nets
PP	Protective woven and non-woven films, tree guards, crates, fertilizer bags
PVC	Mulching, irrigation pipes
PLA	Mulching
PET	Chemical containers
